# (*E*)-3-(3-Chloro­phen­yl)-*N*-(4-hy­droxy-3-meth­oxy­benz­yl)acryl­amide

**DOI:** 10.1107/S1600536810022713

**Published:** 2010-06-18

**Authors:** Liang-You Xia, Wen-Long Wang, Yan-Lan Huang, Shang Shan

**Affiliations:** a Department of Chemistry, Zunyi Normal College, People’s Republic of China; b College of Chemical Engineering and Materials Science, Zhejiang University of Technology, People’s Republic of China

## Abstract

In the title compound, C_17_H_16_ClNO_3_, the 4-hy­droxy-3-meth­oxy­benzyl group is planar [maximum atomic deviation = 0.0138 (16) Å] and is nearly perpendicular to the chloro­benzene ring, making a dihedral angle of 84.67 (4)°. The chloro­benzene and amide groups are located on the opposite sides of the C=C bond, showing an *E* configuration. The relatively long C=O bond distance of 1.2364 (19) Å and the short C—N bond distance of 1.341 (2) Å suggest electron delocalization in the amide fragment. Inter­molecular O—H⋯O, N—H⋯O and weak C—H⋯O hydrogen bonding is present in the crystal structure.

## Related literature

The title compound is a derivative of capsaicin. For the biological activity of capsaicin, see: Kaga *et al.* (1989 ▶). For a related structure, see: Huang *et al.* (2010 ▶). For electron delocal­ization in amide groups, see: Xia *et al.* (2009 ▶).
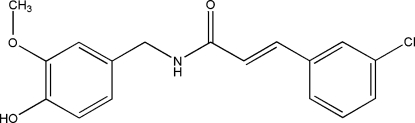


## Experimental

### 

#### Crystal data

C_17_H_16_ClNO_3_
*M* *_r_* = 317.76
Monoclinic, 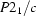

*a* = 9.036 (3) Å
*b* = 14.972 (5) Å
*c* = 11.768 (4) Å
β = 95.047 (5)°
*V* = 1585.9 (9) Å^3^
*Z* = 4
Mo *K*α radiation
μ = 0.25 mm^−1^
*T* = 294 K
0.40 × 0.38 × 0.36 mm

#### Data collection

Rigaku R-AXIS RAPID IP diffractometer
7733 measured reflections
2848 independent reflections
1680 reflections with *I* > 2σ(*I*)
*R*_int_ = 0.034

#### Refinement

*R*[*F* ^2^ > 2σ(*F* ^2^)] = 0.036
*wR*(*F* ^2^) = 0.083
*S* = 0.87
2848 reflections
201 parameters
H-atom parameters constrained
Δρ_max_ = 0.18 e Å^−3^
Δρ_min_ = −0.18 e Å^−3^

### 

Data collection: *PROCESS-AUTO* (Rigaku, 1998 ▶); cell refinement: *PROCESS-AUTO*; data reduction: *CrystalStructure* (Rigaku/MSC, 2002 ▶); program(s) used to solve structure: *SIR92* (Altomare *et al.*, 1993 ▶); program(s) used to refine structure: *SHELXL97* (Sheldrick, 2008 ▶); molecular graphics: *ORTEP-3 for Windows* (Farrugia, 1997 ▶); software used to prepare material for publication: *WinGX* (Farrugia, 1999 ▶).

## Supplementary Material

Crystal structure: contains datablocks I, global. DOI: 10.1107/S1600536810022713/xu2783sup1.cif

Structure factors: contains datablocks I. DOI: 10.1107/S1600536810022713/xu2783Isup2.hkl

Additional supplementary materials:  crystallographic information; 3D view; checkCIF report

## Figures and Tables

**Table 1:** Hydrogen-bond geometry (Å, °)

*D*—H⋯*A*	*D*—H	H⋯*A*	*D*⋯*A*	*D*—H⋯*A*
N1—H1*N*⋯O3^i^	0.86	2.12	2.960 (2)	165
O3—H3*A*⋯O1^ii^	0.82	1.84	2.6491 (18)	172
C2—H2⋯O1^iii^	0.93	2.41	3.298 (2)	161

Symmetry codes: (i) 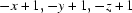; (ii) 
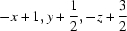
; (iii) 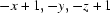.

**Fig. 1.**
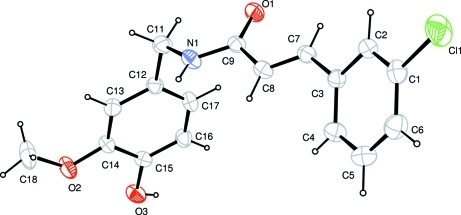
The molecular structure of the title compound with 30% probability displacement (arbitrary spheres for H atoms).

## supplementary crystallographic information

### Comment

The title compound is a derivative of capsaicin, which has been shown a variety of biological activities including mutagenicity (Kaga *et al.* 1989). We prepared the compound recently in the laboratory and determined its crystal structure.

The molecular structure of the title compound is shown in Fig. 1. The chlorobenzene and amide groups are located on the opposite sides of the C7═ C8 bond, showing the E molecular configuration. The hydroxymethoxybenzyl moiety is planar [the maximum atomic deviation being 0.0138 (16) Å for O2 atom], and is nearly perpendicular to the chlorobenzene ring with a dihedral angle of 84.67 (4)°. The dihedral angle between the amide fragment and hydroxymethoxybenzene ring is 89.69 (13)°, which agrees with 85.66 (9)° found in the related derivative of capsaicin, *N*-(4-hydroxy-3-methoxybenzyl)-3-chloro-2,2-dimethylpropanamide (Huang *et al.* 2010). The longer C9═O1 bond distance of 1.2364 (19) Å and the shorter C9—N1 bond distance of 1.341 (2) Å suggest the electron delocalization in the amide fragment, which is comparable to that found in the related compound *N*-(4-Hydroxy-3-methoxybenzyl)benzamide (Xia *et al.* 2009).

Intermolecular O—H···O, N—H···O and weak C—H···O hydrogen bonding are present in the crystal structure (Table 1), which helps to stabilize the crystal structure.

### Experimental

4-Hydroxy-3-methoxy benzylamine HCl salt (4.7 g, 25 mmol) and dimethylformamide (25 ml) were added to a 100 ml 3-necked flask equipped with an additional funnel, a thermometer and a magnetic stirrer. Water solution (10 ml) of NaOH (2.0 g) was added at room temperature. The mixture was stirred at 308 K for 30 min and then cooled to 273 K. An ether solution (10 ml) of 3-(3-chlorophenyl)acryloyl chloride (5.0 g, 25 mmol) was added dropwise at about 273 K over 20 min. After stirred for 2 h at room temperature the mixture was poured into water, and then extracted with ethyl acetate. The ethyl acetate extract was washed with 1 *M* HCl followed by saturated NaHCO_3_ and brine. The extract was then dried over anhydrous Na_2_SO_4_ and filtered. Solvents were removed under vacuum at about 308 K to give a solid crude. Recrystallization was performed twice with an absolute ethyl acetate to obtain single crystals of the title compound.

### Refinement

H atoms were placed in calculated positions with O—H = 0.82, N—H = 0.86 Å, C—H = 0.93–0.97 Å, and refined in riding mode with *U*_iso_(H) = 1.5*U*_eq_(C) for methyl, 1.5*U*_eq_(O) for hydroxy and 1.2*U*_eq_(C,*N*) for the others.

### Crystal data

**Table:**

C_17_H_16_ClNO_3_	*F*(000) = 664
*M**_r_* = 317.76	*D*_x_ = 1.331 Mg m^−^^3^
Monoclinic, *P*2_1_/*c*	Mo *K*α radiation, λ = 0.71073 Å
Hall symbol: -P 2ybc	Cell parameters from 2246 reflections
*a* = 9.036 (3) Å	θ = 2.6–24.8°
*b* = 14.972 (5) Å	µ = 0.25 mm^−^^1^
*c* = 11.768 (4) Å	*T* = 294 K
β = 95.047 (5)°	Prism, colorless
*V* = 1585.9 (9) Å^3^	0.40 × 0.38 × 0.36 mm
*Z* = 4	

### Data collection

**Table:**

Rigaku R-AXIS RAPID IP diffractometer	1680 reflections with *I* > 2σ(*I*)
Radiation source: fine-focus sealed tube	*R*_int_ = 0.034
graphite	θ_max_ = 25.2°, θ_min_ = 3.2°
Detector resolution: 10.0 pixels mm^-1^	*h* = −10→10
ω scans	*k* = −14→17
7733 measured reflections	*l* = −14→12
2848 independent reflections	

### Refinement

**Table:**

Refinement on *F*^2^	Primary atom site location: structure-invariant direct methods
Least-squares matrix: full	Secondary atom site location: difference Fourier map
*R*[*F*^2^ > 2σ(*F*^2^)] = 0.036	Hydrogen site location: inferred from neighbouring sites
*wR*(*F*^2^) = 0.083	H-atom parameters constrained
*S* = 0.87	*w* = 1/[σ^2^(*F*_o_^2^) + (0.0403*P*)^2^] where *P* = (*F*_o_^2^ + 2*F*_c_^2^)/3
2848 reflections	(Δ/σ)_max_ < 0.001
201 parameters	Δρ_max_ = 0.18 e Å^−^^3^
0 restraints	Δρ_min_ = −0.18 e Å^−^^3^

### Special details

**Table:**

Geometry. All e.s.d.'s (except the e.s.d. in the dihedral angle between two l.s. planes) are estimated using the full covariance matrix. The cell e.s.d.'s are taken into account individually in the estimation of e.s.d.'s in distances, angles and torsion angles; correlations between e.s.d.'s in cell parameters are only used when they are defined by crystal symmetry. An approximate (isotropic) treatment of cell e.s.d.'s is used for estimating e.s.d.'s involving l.s. planes.
Refinement. Refinement of *F*^2^ against ALL reflections. The weighted *R*-factor *wR* and goodness of fit *S* are based on *F*^2^, conventional *R*-factors *R* are based on *F*, with *F* set to zero for negative *F*^2^. The threshold expression of *F*^2^ > σ(*F*^2^) is used only for calculating *R*-factors(gt) *etc*. and is not relevant to the choice of reflections for refinement. *R*-factors based on *F*^2^ are statistically about twice as large as those based on *F*, and *R*- factors based on ALL data will be even larger.

### Fractional atomic coordinates and isotropic or equivalent isotropic displacement parameters (Å^2^)

**Table:**

	*x*	*y*	*z*	*U*_iso_*/*U*_eq_	
Cl1	1.00757 (8)	−0.14493 (4)	0.27966 (5)	0.0860 (2)	
N1	0.40981 (17)	0.25930 (9)	0.46714 (12)	0.0486 (4)	
H1N	0.4401	0.2856	0.4085	0.058*	
O1	0.43789 (14)	0.13850 (8)	0.58155 (9)	0.0512 (3)	
O2	0.26985 (16)	0.62811 (8)	0.51708 (10)	0.0635 (4)	
O3	0.44518 (16)	0.63463 (8)	0.70403 (9)	0.0565 (4)	
H3A	0.4801	0.6304	0.7705	0.085*	
C1	0.9795 (2)	−0.03509 (13)	0.32316 (15)	0.0521 (5)	
C2	0.8564 (2)	−0.01564 (12)	0.37947 (14)	0.0466 (5)	
H2	0.7909	−0.0607	0.3961	0.056*	
C3	0.8309 (2)	0.07197 (12)	0.41124 (14)	0.0419 (4)	
C4	0.9333 (2)	0.13733 (13)	0.38823 (14)	0.0514 (5)	
H4	0.9179	0.1962	0.4097	0.062*	
C5	1.0569 (2)	0.11562 (14)	0.33406 (16)	0.0600 (5)	
H5	1.1254	0.1598	0.3201	0.072*	
C6	1.0807 (2)	0.02909 (15)	0.30013 (16)	0.0605 (6)	
H6	1.1637	0.0146	0.2624	0.073*	
C7	0.6954 (2)	0.09312 (12)	0.46534 (13)	0.0434 (5)	
H7	0.6586	0.0499	0.5121	0.052*	
C8	0.6219 (2)	0.16872 (12)	0.45275 (13)	0.0431 (5)	
H8	0.6588	0.2131	0.4077	0.052*	
C9	0.4838 (2)	0.18648 (12)	0.50668 (13)	0.0391 (4)	
C11	0.2816 (2)	0.29694 (12)	0.51673 (16)	0.0534 (5)	
H11A	0.2495	0.2566	0.5742	0.064*	
H11B	0.2003	0.3041	0.4579	0.064*	
C12	0.32141 (19)	0.38656 (11)	0.57021 (14)	0.0424 (4)	
C13	0.2720 (2)	0.46507 (12)	0.51713 (14)	0.0451 (5)	
H13	0.2101	0.4622	0.4498	0.054*	
C14	0.3129 (2)	0.54727 (11)	0.56242 (14)	0.0427 (5)	
C15	0.4062 (2)	0.55182 (11)	0.66299 (13)	0.0414 (4)	
C16	0.4549 (2)	0.47407 (13)	0.71542 (14)	0.0498 (5)	
H16	0.5172	0.4766	0.7826	0.060*	
C17	0.4126 (2)	0.39228 (12)	0.66993 (15)	0.0522 (5)	
H17	0.4462	0.3403	0.7070	0.063*	
C18	0.1775 (3)	0.62875 (14)	0.41375 (17)	0.0868 (8)	
H18A	0.2262	0.5978	0.3560	0.130*	
H18B	0.1584	0.6893	0.3901	0.130*	
H18C	0.0853	0.5996	0.4249	0.130*	

### Atomic displacement parameters (Å^2^)

**Table:**

	*U*^11^	*U*^22^	*U*^33^	*U*^12^	*U*^13^	*U*^23^
Cl1	0.0954 (5)	0.0612 (4)	0.1017 (5)	0.0236 (3)	0.0111 (4)	−0.0185 (3)
N1	0.0612 (11)	0.0352 (9)	0.0511 (9)	0.0042 (8)	0.0150 (8)	0.0023 (7)
O1	0.0566 (9)	0.0501 (8)	0.0473 (7)	−0.0021 (7)	0.0067 (6)	0.0142 (6)
O2	0.0950 (11)	0.0383 (8)	0.0529 (8)	0.0033 (7)	−0.0171 (7)	0.0047 (6)
O3	0.0828 (10)	0.0437 (8)	0.0415 (7)	−0.0120 (7)	−0.0033 (7)	−0.0015 (6)
C1	0.0558 (14)	0.0497 (12)	0.0502 (11)	0.0095 (11)	0.0011 (10)	−0.0019 (9)
C2	0.0452 (12)	0.0418 (11)	0.0520 (11)	−0.0003 (9)	−0.0005 (9)	0.0042 (9)
C3	0.0423 (12)	0.0406 (11)	0.0421 (10)	0.0008 (9)	0.0004 (8)	0.0013 (8)
C4	0.0567 (13)	0.0445 (11)	0.0535 (11)	−0.0041 (11)	0.0076 (10)	−0.0025 (9)
C5	0.0569 (14)	0.0612 (15)	0.0630 (12)	−0.0134 (12)	0.0107 (11)	0.0010 (11)
C6	0.0534 (14)	0.0742 (16)	0.0553 (12)	0.0056 (13)	0.0118 (10)	0.0004 (11)
C7	0.0480 (12)	0.0400 (11)	0.0418 (10)	−0.0041 (10)	0.0020 (8)	0.0026 (8)
C8	0.0542 (13)	0.0345 (11)	0.0412 (9)	−0.0027 (9)	0.0072 (9)	−0.0011 (8)
C9	0.0475 (12)	0.0324 (10)	0.0367 (9)	−0.0034 (9)	−0.0001 (8)	−0.0055 (8)
C11	0.0503 (13)	0.0409 (11)	0.0695 (12)	0.0010 (10)	0.0076 (10)	−0.0041 (10)
C12	0.0408 (11)	0.0360 (11)	0.0516 (11)	0.0038 (9)	0.0095 (9)	−0.0010 (9)
C13	0.0469 (12)	0.0439 (11)	0.0440 (10)	0.0016 (10)	0.0008 (8)	−0.0040 (9)
C14	0.0523 (12)	0.0347 (11)	0.0412 (10)	0.0035 (9)	0.0052 (9)	0.0033 (8)
C15	0.0488 (12)	0.0387 (11)	0.0373 (9)	−0.0045 (9)	0.0079 (8)	−0.0020 (8)
C16	0.0551 (13)	0.0499 (12)	0.0428 (10)	0.0025 (10)	−0.0043 (9)	0.0024 (9)
C17	0.0586 (14)	0.0424 (12)	0.0550 (11)	0.0130 (10)	0.0025 (10)	0.0075 (9)
C18	0.124 (2)	0.0588 (14)	0.0691 (14)	0.0223 (15)	−0.0405 (14)	0.0010 (11)

### Geometric parameters (Å, °)

**Table:**

Cl1—C1	1.747 (2)	C7—C8	1.314 (2)
N1—C9	1.341 (2)	C7—H7	0.9300
N1—C11	1.456 (2)	C8—C9	1.473 (3)
N1—H1N	0.8600	C8—H8	0.9300
O1—C9	1.2364 (19)	C11—C12	1.512 (2)
O2—C14	1.366 (2)	C11—H11A	0.9700
O2—C18	1.413 (2)	C11—H11B	0.9700
O3—C15	1.366 (2)	C12—C17	1.376 (2)
O3—H3A	0.8200	C12—C13	1.386 (2)
C1—C6	1.370 (3)	C13—C14	1.379 (2)
C1—C2	1.375 (3)	C13—H13	0.9300
C2—C3	1.389 (2)	C14—C15	1.393 (2)
C2—H2	0.9300	C15—C16	1.372 (2)
C3—C4	1.389 (2)	C16—C17	1.377 (2)
C3—C7	1.464 (3)	C16—H16	0.9300
C4—C5	1.373 (3)	C17—H17	0.9300
C4—H4	0.9300	C18—H18A	0.9600
C5—C6	1.378 (3)	C18—H18B	0.9600
C5—H5	0.9300	C18—H18C	0.9600
C6—H6	0.9300		
			
C9—N1—C11	124.46 (15)	N1—C9—C8	114.38 (16)
C9—N1—H1N	117.8	N1—C11—C12	110.01 (15)
C11—N1—H1N	117.8	N1—C11—H11A	109.7
C14—O2—C18	117.95 (14)	C12—C11—H11A	109.7
C15—O3—H3A	109.5	N1—C11—H11B	109.7
C6—C1—C2	121.96 (18)	C12—C11—H11B	109.7
C6—C1—Cl1	119.05 (17)	H11A—C11—H11B	108.2
C2—C1—Cl1	118.99 (16)	C17—C12—C13	118.45 (16)
C1—C2—C3	119.33 (18)	C17—C12—C11	120.86 (16)
C1—C2—H2	120.3	C13—C12—C11	120.64 (16)
C3—C2—H2	120.3	C14—C13—C12	121.19 (16)
C2—C3—C4	118.94 (17)	C14—C13—H13	119.4
C2—C3—C7	119.09 (17)	C12—C13—H13	119.4
C4—C3—C7	121.95 (17)	O2—C14—C13	125.63 (15)
C5—C4—C3	120.44 (18)	O2—C14—C15	114.78 (15)
C5—C4—H4	119.8	C13—C14—C15	119.59 (15)
C3—C4—H4	119.8	O3—C15—C16	123.29 (15)
C4—C5—C6	120.7 (2)	O3—C15—C14	117.58 (15)
C4—C5—H5	119.6	C16—C15—C14	119.13 (16)
C6—C5—H5	119.6	C15—C16—C17	120.86 (16)
C1—C6—C5	118.6 (2)	C15—C16—H16	119.6
C1—C6—H6	120.7	C17—C16—H16	119.6
C5—C6—H6	120.7	C12—C17—C16	120.77 (16)
C8—C7—C3	124.84 (17)	C12—C17—H17	119.6
C8—C7—H7	117.6	C16—C17—H17	119.6
C3—C7—H7	117.6	O2—C18—H18A	109.5
C7—C8—C9	123.09 (17)	O2—C18—H18B	109.5
C7—C8—H8	118.5	H18A—C18—H18B	109.5
C9—C8—H8	118.5	O2—C18—H18C	109.5
O1—C9—N1	122.10 (17)	H18A—C18—H18C	109.5
O1—C9—C8	123.52 (16)	H18B—C18—H18C	109.5

### Hydrogen-bond geometry (Å, °)

**Table:**

*D*—H···*A*	*D*—H	H···*A*	*D*···*A*	*D*—H···*A*
N1—H1N···O3^i^	0.86	2.12	2.960 (2)	165
O3—H3A···O1^ii^	0.82	1.84	2.6491 (18)	172
C2—H2···O1^iii^	0.93	2.41	3.298 (2)	161

Symmetry codes: (i) −*x*+1, −*y*+1, −*z*+1; (ii) −*x*+1, *y*+1/2, −*z*+3/2; (iii) −*x*+1, −*y*, −*z*+1.
